# Effect of Prophylactic Radiotherapy on Patients with Stage II-III Esophageal Cancer after Esophageal Cancer Radical Operation and Influencing Factors in Its Recurrence

**DOI:** 10.1155/2021/7462012

**Published:** 2021-08-20

**Authors:** Dan Guo, Kang Zheng

**Affiliations:** ^1^Radiotherapy in One Ward of the Chest, Shanxi Provincial Cancer Hospital, Taiyuan 030013, Shanxi, China; ^2^Special Ward, Shanxi Provincial Cancer Hospital, Taiyuan 030013, Shanxi, China

## Abstract

**Objective:**

To explore the effect of prophylactic radiotherapy on patients with stage II-III esophageal cancer (EC) after esophageal cancer radical operation (ECRO) and influencing factors on EC recurrence.

**Methods:**

Totally, 65 patients with EC in our hospital were enrolled. Among them, 30 patients were treated by routine ECRO as a control group (Con group) and 35 patients by prophylactic radiotherapy as a research group (Res group). Then, the following measures were taken: record the efficacy on both groups, quantify their C-reactive protein (CRP) and white blood cell count (WBC) before and after therapy, evaluate their mental state through the revised piper fatigue scale (PFS-R) before and after therapy, determine their changes in Self-Rating Depression Scale (SDS) and Self-Rating Anxiety Scale (SAS) before and after therapy, compare them in terms of lymph-node metastatic rate (LNMR), hematogenous metastasis rate (HMR), anastomotic recurrence rate (ARR), and 3-year survival rate, compare them in terms of life quality after therapy via the Quality of Life-Core Questionnaire (QLQ-C30), and analyze influencing factors on their recurrence.

**Results:**

The Res group showed a notably higher total effective rate (TER) than the Con group (*P*=0.037). After therapy, CRP and WBC in both groups increased, but their levels were not considerably different in both (*P* > 0.05). Additionally, after therapy, in contrast to the Con group, the Res group got notably lower PFS-R, SDS, and SAS scores, showed notably lower LNMR and ARR and notably higher 3-year survival rate, and experienced notably higher life quality (all *P* < 0.05), and the HMR results were not considerably different in both groups (*P* > 0.05). Moreover, carcinoembryonic antigen (CEA), carbohydrate antigen 125 (CA125), esophageal inflammation history, family medical history, postoperative complications, and lymphatic and vascular infiltration were risk factors for the disease recurrence, and treatment method was the protective factor for it.

**Conclusion:**

For patients with stage II-III EC after ECRO, prophylactic radiotherapy is highly effective and safe and can lower the recurrence rate, so it is worth popularizing in clinical practice.

## 1. Introduction

Esophageal cancer (EC) is a malignancy frequently found in males [1] and also a primary fatal malignancy worldwide [2]. Its primary histological subtypes are squamous cell carcinoma and adenocarcinoma. Obesity, smoking, excessive drinking, and unhealthy eating habits are crucial factors inducing EC [3]. Its symptoms are bound up with its progress. Early EC has no evident symptoms, but middle or late EC is manifested by dysphagia, persistent retrosternal pain, and emaciation [4]. Roughly, EC ranks sixth among all cancers in mortality, gravely threatening patients' lives and safety [5]. Currently, EC is primarily treated based on the principle of individualized comprehensive therapy, including surgery and radiotherapy [6]. Esophageal cancer radical operation (ECRO) is usually selected for patients with early EC, and tumor resection, peripheral lymph-node dissection, and digestive tract reconstruction are adopted [7]. After such therapy, their prognosis is relatively favorable. However, most patients are at the middle/late stage at the time of diagnosis, so they are prone to lymph-node metastasis and thus suffer deterioration [8]. Therefore, in addition to timely surgical therapy, auxiliary radiotherapy is also necessary to improve the efficacy in patients with EC.

According to earlier clinical data, prophylactic radiotherapy can strongly alleviate the deterioration of cancers like small-cell lung cancer and cervical cancer and boost the therapeutic effect against it [9]. For instance, in a study by Nishii et al. [10], prophylactic radiotherapy can lower the complication rate of oral cancer. Roge et al. also pointed out that [11] prophylactic lymph-node radiotherapy is a crucial therapy for early breast cancer. However, its clinical efficacy in EC is rarely studied, and experimental data are insufficient to verify the influence of prophylactic radiotherapy on patients after ECRO and their prognosis. Thus, this study probed into the impact of prophylactic radiotherapy on patients with stage II-III EC after ECRO and influencing factors on EC recurrence, with the aim of offering reliable guidance and potential basis for future clinical therapy of EC.

## 2. Study Design and Treatment

Totally, 65 patients with EC at Shanxi Provincial Cancer Hospital, Taiyuan, Shanxi, PR China, between January 2016 and November 2017 were enrolled. Among them, 30 patients were randomly included in the control group (Con group) treated with routine ECRO, and the remaining 35 patients were included in the research group (Res group) treated with prophylactic radiotherapy. All study participants provided written informed consent. The study was approved by the Ethics Committee of Shanxi Provincial Cancer Hospital, Taiyuan, Shanxi, PR China (20151121), and all experiments conformed to the provisions of the Declaration of Helsinki.

### 2.1. Inclusion and Exclusion Criteria

1. The inclusion criteria: patients confirmed with stage II-III EC via examinations in pathology, laboratory examination, and imaging in our hospital, patients with detailed case data, and those consenting to cooperate with the study
2. The exclusion criteria: patients with other comorbid malignancies, patients with liver or kidney dysfunction, patients with a surgical contraindication, coagulant function abnormality, or immune deficiency, lactating women, pregnant women, referred patients, and those with poor compliance

### 2.2. Methods

All patients were given ECRO. Firstly, in a cutting direction selected according to the pathological tissue of the patient, the cancerous tissue tumors were resected and the surrounding lymph nodes that can be cleaned were cleaned under a maximized operation field or visual field. The proper digestive tract function of the patient after surgery should be ensured, so the patient was given enteral and parenteral nutrition support while he was required to take liquid food as staple food so that he can get faster recovery. Patients in the Res group were given prophylactic radiotherapy four weeks after surgery. Each patient was irradiated after a target radiotherapy area was selected according to his situation. Specifically, single anterior-field radiotherapy was adopted at a dosage of 40 Gy 20 times during the first four weeks, and then the horizontal fields on both sides were irradiated at 20 Gy 10 times during the next 2 weeks and 60 Gy 30 times during the next 6 weeks.

### 2.3. Outcome Measures

The efficacy in the two groups after therapy was recorded. The efficacy was classified into complete remission, partial remission, no change, and progression. Total effective rate (TER) = complete remission rate + partial remission rate [12]. Then, before and after therapy, the C-reactive protein (CRP) and white blood cell count (WBC) in the two groups were quantified and their mental state was evaluated through the revised piper fatigue scale (PFS-R) [13]. Additionally, the psychological changes of the two groups were evaluated using the Self-Rating Depression Scale (SDS) and Self-Rating Anxiety Scale (SAS), and the lymph-node metastatic rate (LNMR), hematogenous metastasis rate (HMR), anastomotic recurrence rate (ARR), and 3-year recurrence rate were compared between them. Moreover, the life quality of both groups was evaluated via the EORTC Quality of Life Questionnaire (QLQ-C30), and influencing factors on the disease recurrence were analyzed.

### 2.4. Statistical Analyses

In this study, SPSS22.0 was used for data processing and GraphPad7 for visualization of data into corresponding figures. Intergroup comparison of enumeration data, presented by (%), was carried out via the chi-square test, while intergroup and multigroup comparisons of measurement data, presented as the mean ± SD, were conducted via the *t*-test and one-way ANOVA, respectively, and the LSD post hoc test was conducted. Additionally, data at multiple times were analyzed via the repeated measures analysis of variance and Bonferroni post hoc test. *P* < 0.05 denotes a notable difference.

## 3. Results

### 3.1. Baseline Data of Patients

The two groups were not considerably different in age, body mass index (BMI), sex, course of disease, living environment, smoking history, drinking history, and nationality (all *P* > 0.05) (Table 1).

### 3.2. Efficacy in the Two Groups

From the comparison of efficacy between the two groups, the Res group showed a TER of 85.71% (30 patients), with complete remission in 19 patients (54.29%), partial remission in 11 patients (31.43%), no change in 4 patients (11.43%), and progression in 1 patient (2.86%), while the Con group showed a TER of 63.33% (19 patients), with complete remission in 7 patients (23.33%), partial remission in 12 patients (40.00%), no change in 6 patients (20.00%), and progression in 5 patients (16.67%), so the TER in the Res group was notably higher (*p* = 0.037) (Table 2).

### 3.3. Changes of CRP and WBC in the Two Groups

**Figure 1:**
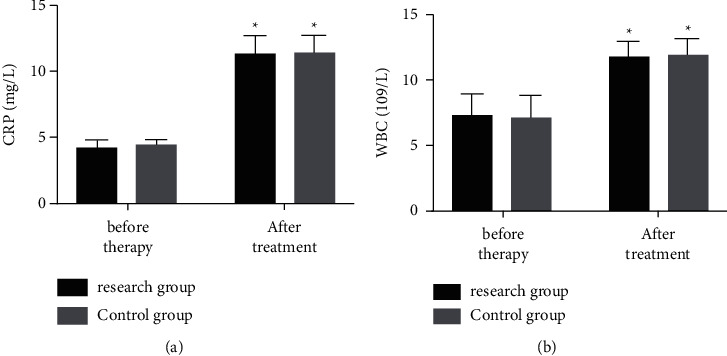
Changes of CRP and WBC. Pretherapy and posttherapy (a) CRP and (b) WBC of the two groups. The symbol *∗* signifies vs. the situation before treatment.

According to quantification of CRP and WBC in the two groups before and after therapy, after therapy, both groups showed notably increased levels of CRP and WBC (both *P* < 0.05), but in both groups, the levels were not significantly different (*P* > 0.05) (Figure 1).

### 3.4. PFS-R Scores of the Two Groups

**Figure 2:**
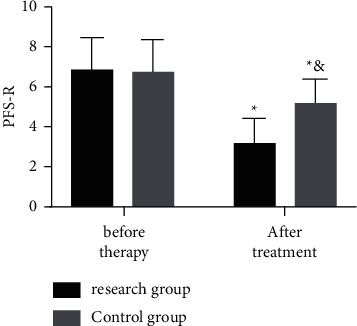
Pretherapy and posttherapy PFS-R scores of the two groups. The symbol *∗* signifies vs. the situation before treatment; &, vs. the Res group.

The comparison of mental state between the two groups via PFS-R before and after therapy showed that, before therapy, the two groups were not considerably different in PFS-R score (*P* > 0.05), while after therapy, the score of both groups declined and the decline in the Res group was more notable (*P* < 0.05) (Figure 2).

### 3.5. Psychological Changes of the Two Groups

**Figure 3:**
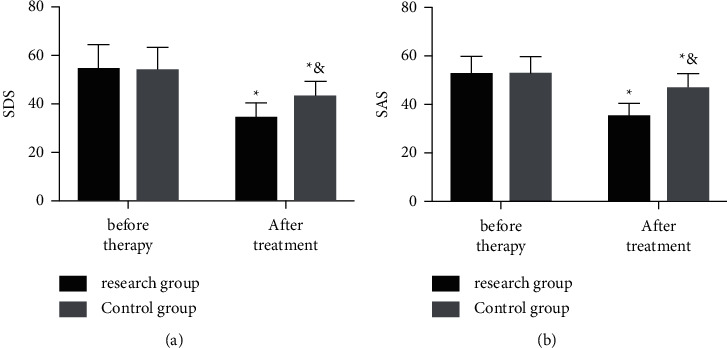
Psychological changes of the two groups before and after treatment. Pretherapy and posttherapy (a) SDS and (b) SAS of the two groups. The symbol *∗* signifies vs. the situation before treatment; &, vs. the Res group.

According to the comparison of the two groups in alleviation of mental depression and anxiety before and after therapy, before therapy, the two groups were not significantly different in SDS and SAS scores (both *P* > 0.05), while after therapy, SDS and SAS scores of both groups declined and the scores of the Res group were low (all *P* < 0.05) (Figure 3).

### 3.6. Incidence of Posttherapy Complications

According to a comparison of LNMR, HMR, ARR, and 3-year survival rate between the two groups after therapy, the Res group presented notably lower LNMR and ARR and considerably higher 3-year survival rate and the HMR results were not considerably different in both groups (all *P* < 0.05) (Table 3).

### 3.7. Posttherapy Life Quality of the Two Groups

**Figure 4:**
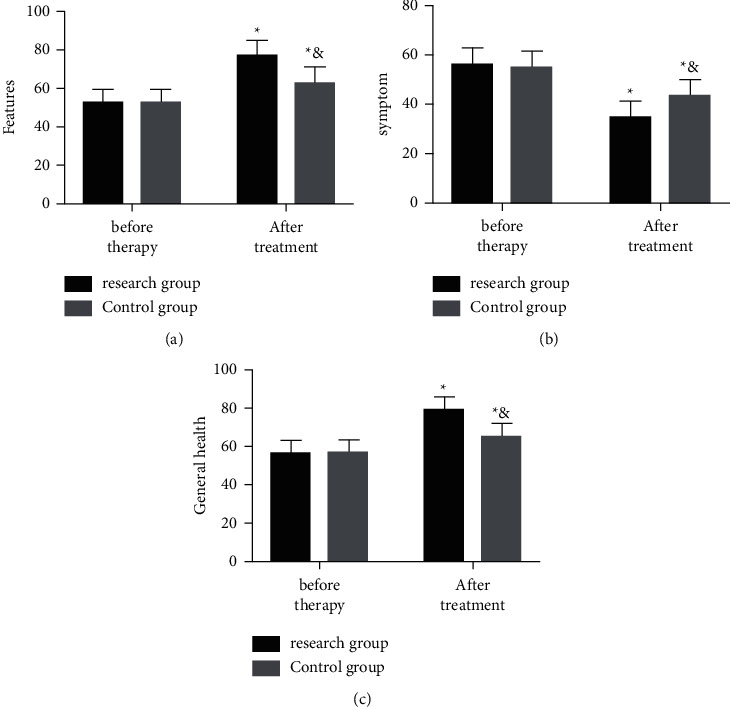
Posttherapy life quality of the two groups. Posttherapy (a) functional scores, (b) symptom scores, and (c) general health scores of the two groups. The symbol *∗* signifies vs. *∗* the situation before treatment; &, the Res group.

The posttherapy life quality of the two groups was evaluated via the functional, symptom, and general health subscales. As a result, after therapy, the functional and general health scores of the two increased and the scores of the Res group were notably higher than those of the Con group (both *P* > 0.05). Additionally, after therapy, the symptom scores of both groups decreased, and the score of the Res group was notably lower (both *P* < 0.05) (Figure 4).

### 3.8. Influencing Factors of Disease Recurrence

Logistic regression analysis on influencing factors of disease recurrence showed that carcinoembryonic antigen (CEA), carbohydrate antigen 125 (CA125), esophageal inflammation history, family medical history, postoperative complications, and lymphatic and vascular infiltration were risk factors for the disease recurrence, and the treatment method was the protective factor (Tables 4 and 5).

## 4. Discussion

EC is the most prevalent gastrointestinal cancer [14]. Its morbidity and mortality have been brought under control, but it is still a serious threat to the life and health of the Chinese due to its various pathogenic factors [15]. Radical surgery is the primary therapy for EC [16], but the selection of adjuvant treatments such as radiotherapy after ECRO is still controversial in clinical practice. For improving the survival rate of Chinese people with EC, this study probed into the effect of prophylactic radiotherapy on patients with stage II-III EC after ECRO and influencing factors on EC recurrence.

Before the study, we collected baseline data of the two groups and found no notable difference between them in age, BMI, sex, course of disease, living environment, smoking history, drinking history, and nationality, which suggests their comparability. Firstly, we evaluated the efficacy in the two groups. The efficacy was classified into complete remission, partial remission, no change, and progression. As our results showed, the Res group presented a notably higher TER than the Con group (85.71% (30 patients) vs. 63.33% (19 patients)). The data imply that prophylactic radiotherapy can contribute to stronger clinical efficacy in patients with EC, with a beneficial influence on their clinical symptoms and survival rate. According to associated references, Sun et al. [17] pointed out the remarkable efficacy of prophylactic radiotherapy in patients with nasopharyngeal carcinoma. There are also studies which support the idea that prophylactic radiotherapy can strongly prevent recurrence and metastasis of tumor diseases [18]. These conclusions can verify the results of our study. The tumor of patients with middle or late EC is large, so the postoperative effect is inconsistent due to the difference of their tumor location or tumor size, and prophylactic radiotherapy can further kill the residual tumor, which may be one major reason for the efficacy improvement by prophylactic radiotherapy. We quantified CRP and WBC in the two groups and found after therapy that both groups showed notably increased CRP and WBC, but the levels were not considerably different in both groups, which were in line with results discovered previously [19]. We reason that for patients with EC, after surgery or postoperative radiotherapy, their body will produce stress response and their CRP and WBC will increase. At the same time, the two groups showed no notable difference in CRP and WBC after therapy, which further reflected the effectiveness and safety of prophylactic radiotherapy. One study by Kugele et al. [20] has pointed out that prophylactic radiotherapy can strongly enhance local control and survival among patients undergoing radical mastectomy. It is similar to our study result, which supports the conclusion that prophylactic radiotherapy can deliver a strong inhibitory influence on postoperative tumor recurrence. We also adopted PFS-R for analyzing and comparing the mental state of the two groups. As the results showed, before therapy, the two groups were not greatly different in PFS-R score, while after therapy, the scores of both groups declined and the decline in the Res group was more notable. The results imply the crucial role of prophylactic radiotherapy in alleviating the fatigue state of patients and improving their life quality. Cancer-associated fatigue is a prevalent clinical symptom of EC. Diseases take a serious toll on patients' mental and physical strength, which is prone to aggravating their negative emotions and increasing the difficulty of treating muscle diseases [21]. Prophylactic radiotherapy may greatly alleviate patients' clinical symptoms, which is the primary task to improve their life quality [22]. We compared the two groups for alleviation of mental depression and anxiety before and after therapy and found that before therapy, the two groups were not significantly different in SDS and SAS scores, while after therapy, SDS and SAS scores of both groups declined and the scores of the Res group were low. The results further demonstrate the favorable clinical efficacy of prophylactic radiotherapy because of its function in strongly alleviating patients' symptoms and unhealthy emotions. Moreover, we compared the LNMR, HMR, ARR, and 3-year survival rates of the two groups after therapy, finding that the Res group presented notably lower LNMR and ARR and notably higher 3-year survival rate, but they were not considerably different in HMR. The acquired data reflect the safety and effectiveness of prophylactic radiotherapy in the Res group from one angle and further reflect the reason for the higher survival rate in the Res group. The data also confirm the crucial clinical value of prophylactic radiotherapy after ECRO. We evaluated the posttherapy life quality of the two groups via the functional, symptom, and general health subscales. As a result, after therapy, the functional and general health scores of the two increased and the scores of the Res group were notably higher than those of the Con group. Additionally, after therapy, the symptom scores of both groups decreased and the score of the Res group was notably lower. The results were in step with our above analysis. Finally, we carried out logistic regression analysis on influencing factors of disease recurrence, finding that CEA, CA125, esophageal inflammation history, family medical history, postoperative complications, and lymphatic and vascular infiltration were risk factors for the disease recurrence and postoperative prophylactic radiotherapy was the protective factor for it. Thus, postoperative prophylactic radiotherapy is pivotal for patients. The study has indicated that low-dose, prophylactic, extended-field, intensity-modulated radiotherapy combined with cisplatin could effectively improve the prognosis of patients with cervical cancer [23].

However, due to the short experimental period, we were unable to evaluate the long-run prognosis of patients, and some experimental results may not be highly representative because of the small sample size of included subjects. It provides potential basis for further experimental analysis. In addition, it is required to understand the exact mechanism of prophylactic radiotherapy through basic experiments to provide more perfect clinical references.

To sum up, for patients with stage I-II EC after ECRO, prophylactic radiotherapy is highly effective and safe and can lower the recurrence rate, so it is worth popularizing in clinical practice.

## Figures and Tables

**Table 1:** Comparison of clinical baseline data.

	The Res group (*n* = 35)	The Con group (*n* = 30)	*χ*2 or *t*/*P*
*Age (Y)*	58.6 ± 5.4	59.3 ± 5.2	0.530/0.598
*BMI (kg/cm*^*2*^)	24.2 ± 1.4	24.5 ± 1.7	0.780/0.438
*Sex*			0.002/0.968
Male	22 (62.86%)	19 (63.33%)	
Female	13 (37.14%)	11 (36.67%)	

*Course of disease (month)*	15.2 ± 2.3	15.6 ± 2.5	0.672/0.504
*Residential environment*			0.094/0.758
Urban area	20 (57.14%)	16 (53.33%)	
Rural area	15 (42.86%)	14 (46.67%)	

*Smoking history*			0.511/0.475
Yes	28 (80.00)	26 (86.67)	
No	7 (20.00)	4 (13.33)	

*Drinking history*			0.275/0.600
Yes	30 (85.71)	27 (90.00)	
No	5 (14.29)	3 (10.00)	

*Nationality*			2.407/0.121
Han nationality	35 (100.00%)	28 (93.33%)	
Minority nationality	0 (0.00%)	2 (6.67%)	

**Table 2:** Efficacy in the two groups.

	The Res group (*n* = 35)	The Con group (*n* = 30)	*χ*2	*P* value
Complete remission	19 (54.29)	7 (23.33)		
Partial remission	11 (31.43)	12 (40.00)		
No change	4 (11.43)	6 (20.00)		
Progress	1 (2.86)	5 (16.67)		
Total effective rate (%)	30 (85.71)	19 (63.33)	4.361	0.037

**Table 3:** Prognosis of the patients after therapy.

	The Res group (*n* = 35)	The Con group (*n* = 30)	*χ*2	*P* value
Lymph-node metastatic rate %	3 (8.57)	9 (30.00)	4.928	0.026^*∗*^
Hematogenous metastasis rate %	3 (8.57)	4 (13.33)	0.381	0.537
Anastomotic recurrence rate %	2 (5.71)	7 (23.33)	4.204	0.040^*∗*^
3-year survival rate %	25 (71.43)	14 (46.67)	4.127	0.042^*∗*^

^∗^meant that the differences of the two groups were significant.

**Table 4:** The information of assignment.

Assignment	
CEA	Raw data were used for analysis
CA125	Raw data were used for analysis
Esophageal inflammation history	0 was assigned to No and 1 to Yes
Family medical history	0 was assigned to No and 1 to Yes
Postoperative complications	0 was assigned to No and 1 to Yes
Lymphatic vessel invasion	0 was assigned to No and 1 to Yes
Treatment methods	0 was assigned to postoperative prophylactic radiotherapy and 1 to no postoperative prophylactic radiotherapy

**Table 5:** Univariate and multivariate analysis on influencing factors of disease recurrence.

	Univariate			Multivariate		
	OR	95% CI	*P* value	OR	95% CI	*P* value
CEA	15.63	4.63–26.63	0.019	4.63	2.54–9.63	0.021
CA125	12.6063	7.63–13.41	0.029	6.63	2.63–12.63	0.018
Esophageal inflammation history	2.63	1.16–3.84	0.019	1.63	0.54–2.63	0.187
Family medical history	1.33	0.42–3.63	0.184	—	—	—
Postoperative complications	1.63	0.23–4.63	0.002	1.14	0.18–1.63	0.006
Lymphatic vessel invasion	1.85	0.15–3.63	0.009	1.56	0.06–1.92	0.001
Treatment methods	0.42	0.06–1.06	0.004	0.75	0.12–1.15	0.002

## Data Availability

All data are available from the corresponding author upon request.
